# Supplementary figures and images for: Effect of any form of steroids in comparison with that of other medications on the duration of olfactory dysfunction in patients with COVID-19: A systematic review of randomized trials and quasi-experimental studies
Source: PLoS One. 2023 Aug 2;18(8):e0288285. doi: 10.1371/journal.pone.0288285 (PMC10395913; doi:10.1371/journal.pone.0288285)

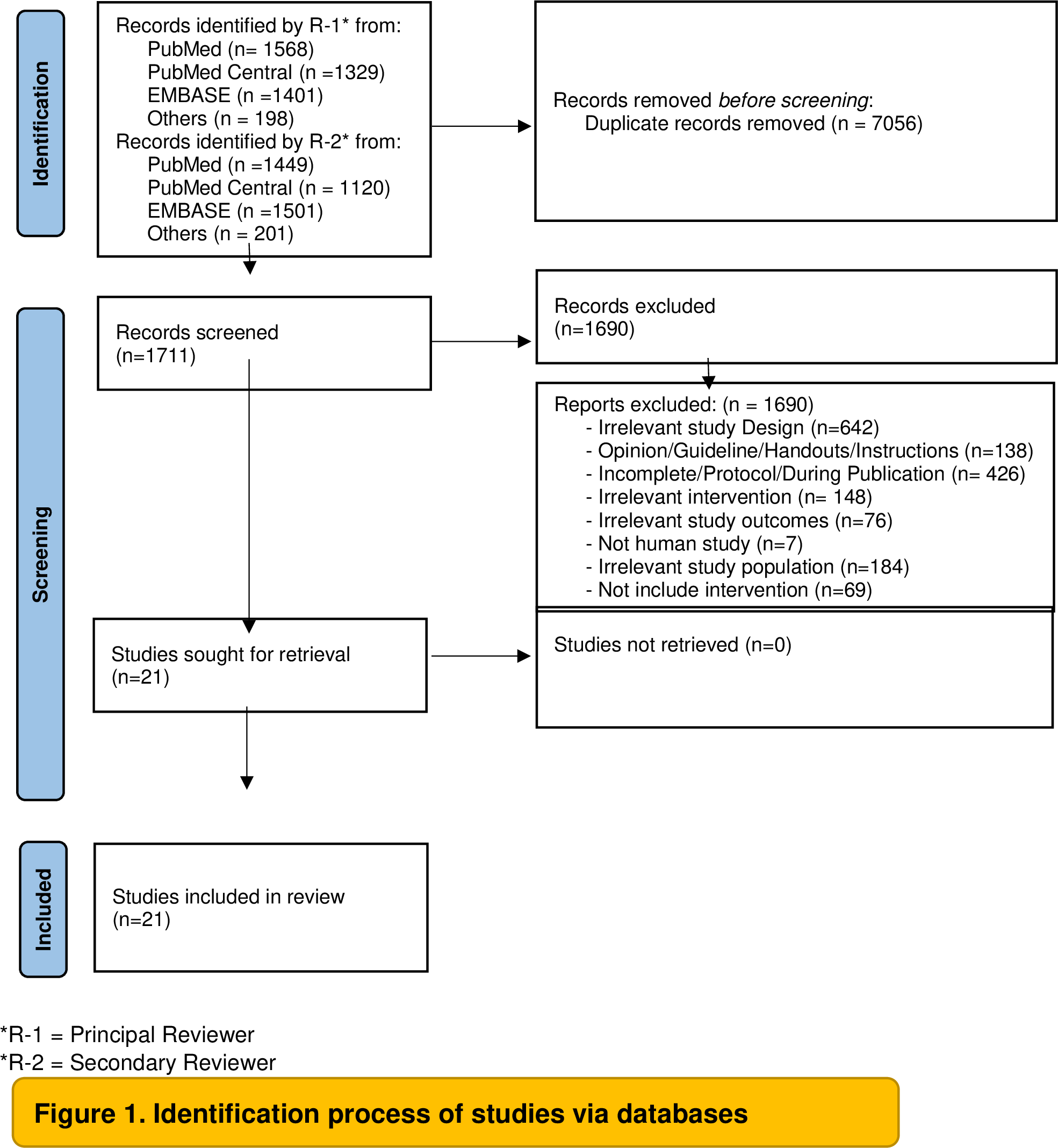

Supplement: S1 Fig — (TIF) [file pone.0288285.s001.tif]
