# Supplementary material for: Effect of any form of steroids in comparison with that of other medications on the duration of olfactory dysfunction in patients with COVID-19: A systematic review of randomized trials and quasi-experimental studies
Source: PLoS One. 2023 Aug 2;18(8):e0288285. doi: 10.1371/journal.pone.0288285 (PMC10395913; doi:10.1371/journal.pone.0288285)
Supplement: S1 Table — (DOCX) [file pone.0288285.s002.docx]

**PRISMA 2020 CHECKLIST**

| **Section and Topic** | **Item #** | **Checklist item** | **Location where item is reported** |
| --- | --- | --- | --- |
| **TITLE** | | |  |
| Title | 1 | Identify the report as a systematic review. | Under the sub-heading "Study Design" of "Material and Method" p.5 |
| **ABSTRACT** | | |  |
| Abstract | 2 | See the PRISMA 2020 for Abstracts checklist. | Mentioned in the section "Abstract" p.2 |
| **INTRODUCTION** | | |  |
| Rationale | 3 | Describe the rationale for the review in the context of existing knowledge. | Under the heading "Introduction" p.3 |
| Objectives | 4 | Provide an explicit statement of the objective(s) or question(s) the review addresses. | Under the heading "Introduction" p.3 |
| **METHODS** | | |  |
| Eligibility criteria | 5 | Specify the inclusion and exclusion criteria for the review and how studies were grouped for the syntheses. | Under the sub-headings "Study Design" p.5 and “Typical Inclusion Criteria” p.6 of "Material and Method" p.5 |
| Information sources | 6 | Specify all databases, registers, websites, organisations, reference lists and other sources searched or consulted to identify studies. Specify the date when each source was last searched or consulted. | Under the sub-heading "Information Sources and Searching Strategy” p.8 of "Material and Method" |
| Search strategy | 7 | Present the full search strategies for all databases, registers and websites, including any filters and limits used. | Under the sub-heading "Information Sources and Searching Strategy” p.8 of "Material and Method" |
| Selection process | 8 | Specify the methods used to decide whether a study met the inclusion criteria of the review, including how many reviewers screened each record and each report retrieved, whether they worked independently, and if applicable, details of automation tools used in the process. | Under the sub-heading "Details of Selection Process” p.11 of "Material and Method" |
| Data collection process | 9 | Specify the methods used to collect data from reports, including how many reviewers collected data from each report, whether they worked independently, any processes for obtaining or confirming data from study investigators, and if applicable, details of automation tools used in the process. | Under the sub-heading "Data Extraction and Analysis” p.12 of "Material and Method" |
| Data items | 10a | List and define all outcomes for which data were sought. Specify whether all results that were compatible with each outcome domain in each study were sought (e.g. for all measures, time points, analyses), and if not, the methods used to decide which results to collect. | The operational definitions of the variable in the data extraction sheet and details of the extraction process were specified in Table 2 p.10 under the heading" of "Material and Method"  Under the sub-headings "Intervention” p.7 “Control group” p.8 and “Outcomes” p.8 of "Material and Method" |
|  | 10b | List and define all other variables for which data were sought (e.g. participant and intervention characteristics, funding sources). Describe any assumptions made about any missing or unclear information. | The operational definitions of the variable in the data extraction sheet and details of the extraction process were specified in Table 2 p.10 under the heading" of "Material and Method"  Under the sub-headings "Intervention” p.7 “Control group” p.8 and “Outcomes” p.8 of "Material and Method" |
| Study risk of bias assessment | 11 | Specify the methods used to assess the risk of bias in the included studies, including details of the tool(s) used, how many reviewers assessed each study and whether they worked independently, and if applicable, details of automation tools used in the process. | Under the sub-heading "Critical Appraisal Tools" p.12 of "Material and Method" and the heading of "Strengths and Limitations of Study" p.32 |
| Effect measures | 12 | Specify for each outcome the effect measure(s) (e.g. risk ratio, mean difference) used in the synthesis or presentation of results. | Under the sub-headings “Intervention” p.7, “Control Group” p.8, “Outcomes” p.8, and " Information Sources and Searching Strategy " p.8 of "Material and Method" |
| Synthesis methods | 13a | Describe the processes used to decide which studies were eligible for each synthesis (e.g. tabulating the study intervention characteristics and comparing against the planned groups for each synthesis (item #5)). | Under the sub-heading "Data Extraction and Analysis" p.12 of "Material and Method" |
|  | 13b | Describe any methods required to prepare the data for presentation or synthesis, such as handling of missing summary statistics, or data conversions. | Under the sub-heading "Data Extraction and Analysis" p.12 of "Material and Method" |
|  | 13c | Describe any methods used to tabulate or visually display results of individual studies and syntheses. | Under the sub-heading "Data Extraction and Analysis" p.12 of "Material and Method" |
|  | 13d | Describe any methods used to synthesize results and provide a rationale for the choice(s). If meta-analysis was performed, describe the model(s), method(s) to identify the presence and extent of statistical heterogeneity, and software package(s) used. | Under the sub-heading "Data Extraction and Analysis" p.12 of "Material and Method" |
|  | 13e | Describe any methods used to explore possible causes of heterogeneity among study results (e.g. subgroup analysis, meta-regression). | Under the sub-heading "Data Extraction and Analysis" p.12 of "Material and Method" |
|  | 13f | Describe any sensitivity analyses conducted to assess robustness of the synthesized results. | Not applicable |
| Reporting bias assessment | 14 | Describe any methods used to assess risk of bias due to missing results in a synthesis (arising from reporting biases). | Under the sub-headings "Data Extraction and Analysis" p.12 of "Material and Method" and the heading of "Strengths and Limitations of Study" p.32 |
| Certainty assessment | 15 | Describe any methods used to assess certainty (or confidence) in the body of evidence for an outcome. | Not applicable |
| **RESULTS** | | |  |
| Study selection | 16a | Describe the results of the search and selection process, from the number of records identified in the search to the number of studies included in the review, ideally using a flow diagram. | Under the sub-heading "Details of Selection Process" p.13 of "Results" |
|  | 16b | Cite studies that might appear to meet the inclusion criteria, but which were excluded, and explain why they were excluded. | Under the sub-heading "Details of Selection Process" p.13 of "Results" |
| Study characteristics | 17 | Cite each included study and present its characteristics. | Under the sub-heading "Characteristics of the included Studies" p.14 of "Results" and in Table 3 p.14 |
| Risk of bias in studies | 18 | Present assessments of risk of bias for each included study. | Under the sub-heading "Reporting Quality of the Included Studies" p.16 of "Results" and in Table 4 p.16 and 5 p.17 |
| Results of individual studies | 19 | For all outcomes, present, for each study: (a) summary statistics for each group (where appropriate) and (b) an effect estimate, and its precision (e.g. confidence/credible interval), ideally using structured tables or plots. | The results extracted from the selected studies were displayed in "Table 6 Interventions and their effects on the Covid-19 related olfactory dysfunction" p.18 and under the sub-heading “Characteristics of the included Studies" p.14 |
| Results of syntheses | 20a | For each synthesis, briefly summarise the characteristics and risk of bias among contributing studies. | It was described in "Table 6 Interventions and their effects on the Covid-19 related olfactory dysfunction" p.18 |
|  | 20b | Present results of all statistical syntheses conducted. If meta-analysis was done, present for each the summary estimate and its precision (e.g. confidence/credible interval) and measures of statistical heterogeneity. If comparing groups, describe the direction of the effect. | It was described in "Table 6  Interventions and their effects on the Covid-19 related olfactory dysfunction" p.18 |
|  | 20c | Present results of all investigations of possible causes of heterogeneity among study results. | Details of results are synthesized in “Interventional Treatments for Olfactory Dysfunction” p.18 of “Results” |
|  | 20d | Present results of all sensitivity analyses conducted to assess the robustness of the synthesized results. | Details of results are synthesized in “Results” p.13 |
| Reporting biases | 21 | Present assessments of risk of bias due to missing results (arising from reporting biases) for each synthesis assessed. | The assessment of the reporting bias was presented under the sub-heading "Reporting Quality of the Included Studies" p.15 of "Results" |
| Certainty of evidence | 22 | Present assessments of certainty (or confidence) in the body of evidence for each outcome assessed. | Not Applicable |
| **DISCUSSION** | | |  |
| Discussion | 23a | Provide a general interpretation of the results in the context of other evidence. | Under the sub-heading "Discussion" p.26 |
|  | 23b | Discuss any limitations of the evidence included in the review. | Under the heading "Strengths and Limitation of Study" p.32 |
|  | 23c | Discuss any limitations of the review processes used. | Under the heading "Strengths and Limitation of Study" p.32 |
|  | 23d | Discuss implications of the results for practice, policy, and future research. | Under the heading of "Conclusion" p.32 |
| **OTHER INFORMATION** | | |  |
| Registration and protocol | 24a | Provide registration information for the review, including register name and registration number, or state that the review was not registered. | Not Applicable |
|  | 24b | Indicate where the review protocol can be accessed, or state that a protocol was not prepared. | Not Applicable |
|  | 24c | Describe and explain any amendments to the information provided at registration or in the protocol. | Not Applicable |
| Support | 25 | Describe sources of financial or non-financial support for the review, and the role of the funders or sponsors in the review. | Not Applicable |
| Competing interests | 26 | Declare any competing interests of review authors. | Not Applicable |
| Availability of data, code and other materials | 27 | Report which of the following are publicly available and where they can be found: template data collection forms; data extracted from included studies; data used for all analyses; analytic code; any other materials used in the review. | Available via direct contact of the corresponding author |
