# Supplementary material for: Effect of any form of steroids in comparison with that of other medications on the duration of olfactory dysfunction in patients with COVID-19: A systematic review of randomized trials and quasi-experimental studies
Source: PLoS One. 2023 Aug 2;18(8):e0288285. doi: 10.1371/journal.pone.0288285 (PMC10395913; doi:10.1371/journal.pone.0288285)
Supplement: S2 Table — (DOCX) [file pone.0288285.s003.docx]

| **Table 1. Objectives and measurement tools of the included studies** | | |
| --- | --- | --- |
| **Study Titles** | **Study Objectives** | **Measurement Tools** |
| A new strategy for the treatment of Anosmia and Ageusia in COVID-19 patients | To discover the effect of coffee on the taste and smell dysfunction following Covid-19 | Duration for smell recovery |
| Olfactory Training and Visual Stimulation Assisted by a Web Application for Patients with Persistent Olfactory Dysfunction After SARS-CoV-2 Infection: Observational Study | To determine the effect of olfactory training (visual stimulation) in Covid-19 patients with anosmia for more than one month | 11-point visual analogue scale (VAS) |
| Effect of Lavender (Lavandula angustifolia L.) syrup on olfactory dysfunction in COVID-19 infection: A pilot controlled clinical trial | To determine the effect of lavender syrup on Covid-19 induced olfactory dysfunction in outpatients | 11-point visual analogue scale (VAS) |
| Corticosteroid nasal spray for recovery of smell sensation in COVID-19 patients: A randomized controlled trial | To know the effectiveness of mometasone furoate nasal spray for persistent anomia following Covid-19 | online questionnaire |
| Effect of nasal corticosteroid in the treatment of anosmia due to COVID-19: A randomized double-blind placebo-controlled study | To identify the therapeutic effect of betamethasone nasal drops in Covid-19 anosmia | 11-point visual analogue scale (VAS) |
| Efficacy and safety of oral corticosteroids and olfactory training in the management of COVID‐19‐related loss of smell | To determine the effect of oral corticosteroids and olfactory training in Covid-19 patients with anosmia | Duration for smell recovery |
| Mometasone furoate nasal spray in the treatment of patients with COVID-19 olfactory dysfunction: A randomized, double-blind clinical trial | To discover the efficacy of mometasone nasal spray in Covid-19 patients with severe microsomia or anosmia | Sniffin Sticks Test, TDI scores |
| Olfactory Disturbances as Presenting Manifestation Among Egyptian Patients with COVID-19: Possible Role of Zinc | To study the serum zinc level in Covid-19 patients and to determine the usage of zinc therapy in patients with olfactory dysfunction | 11-point visual analogue scale (VAS) and University of Pennsylvania Smell Identification Test (UPSIT) - adapted as Iran-SIT |
| Persistent Parosmia Caused By COVID-19 Infection: An Emerging Symptom | To study the clinical symptoms of post-Covid-19 parosmia and to determine the effectiveness of various intervention methods | Duration for smell recovery |
| The randomized clinical trial “olfactory dysfunction after COVID-19: olfactory rehabilitation therapy vs. intervention treatment with Palmitoylethanolamide and Luteolin”: preliminary results | To determine the effect of Palmitoyleth- ethanolamide (PEA) and Luteolin in the recovery of olfactory function in Covid-19 patients | Modified Arabic 20- Items Sino-Nasal Outcome Test (MA-SNOT-20) |
| Short-Term Efficacy and Safety of Oral and Nasal Corticosteroids in COVID-19 Patients with Olfactory Dysfunction: A European Multicenter Study | To compare the effectiveness and safety of oral or nasal corticosteroid plus olfactory training with olfactory training alone for treatment of Covid-19 related anosmia | Sniffin Sticks Test, TDI scores |
| The outcome of fluticasone nasal spray on anosmia and triamcinolone oralpaste in dysgeusia in COVID-19 patients | To know the effect of fluticasone nasal spray on anosmia and triamcinolone oral paste on dysgeusia in Covid-19 patients | Sniffin Sticks Test, I scores |
| Comparison of the Healing Effect of Nasal Saline Irrigation with Triamcinolone Acetonide Versus Nasal Saline Irrigation alone in COVID-19 Related Olfactory Dysfunction: A Randomized Controlled Study | To compare the effects of nasal saline irrigation plus nasal triamcinolone spray vs nasal saline irrigation alone on Covid-19 anosmia | Subjective smell assessment by using 5 different smells |
| Modified Olfactory Training Is an Effective Treatment Method for COVID-19 Induced Parosmia | To retrospectively discover the effect of modified olfactory training on Covid-19 parosmia | Self-Rating Olfactory Score (SROS), and Olfactory Dysfunction Duration (ODD) |
| Inflawell improves neutrophil‑to‑lymphocyte ratio and shortens hospitalization in patients with moderate COVID‑19, in a randomized double-blind placebo-controlled clinical trial | To determine the effect of Inflawell syrup, a Boswellia extract formulation, on the disease severity of Covid-19 | Sniffin Sticks Test, TDI scores |
| Omega-3 supplementation in post-viral olfactory dysfunction: a pilot study | To investigate the effect of Omega-3 supplementation on Covid-19 anosmia | Physical examination and Lab investigation |
| Post-Vaccination SARS-CoV-2 Infections among Health Workers at the University Hospital of Verona, Italy: A Retrospective Cohort Survey | To determine the effects of Covid-19 vaccination | Sniffin Sticks Test, TDI scores |
| The Role of social media in Improving Patient Recruitment for Research Studies on Persistent Post-Infectious Olfactory Dysfunction | To assess the desire of the patients with post-infectious olfactory dysfunction to participate in research | ad hoc semi-structured questionnaire about the prevalence and duration of symptoms |
| Association of Vaccination with the Persistence of Post-COVID Symptoms | To compare the post covid symptoms between vaccinated and unvaccinated subjects | 11-point visual analogue scale (VAS) |
| Post‐COVID‐19 olfactory dysfunction: carbamazepine as a treatment option in a series of cases | To study the effectiveness of carbamazepine on the treatment of post-Covid-19 olfactory dysfunction | Single-item 5-point Likert question (1 = no sense of smell to 5 = excellent sense of smell) adapted from the PhenX Toolkit |
| New Onset of Smell and Taste Loss Are Common Findings Also in Patients with Symptomatic COVID-19 After Complete Vaccination | To discover the clinical presentation of Covid-19 after full vaccination | MRI, questioning each patient regarding changes or improvement in their olfactory ability |
