# Supplementary material for: Effect of any form of steroids in comparison with that of other medications on the duration of olfactory dysfunction in patients with COVID-19: A systematic review of randomized trials and quasi-experimental studies
Source: PLoS One. 2023 Aug 2;18(8):e0288285. doi: 10.1371/journal.pone.0288285 (PMC10395913; doi:10.1371/journal.pone.0288285)
Supplement: Attachment — Submitted filename: 1-PLOS ONE-Reviewer Comments.docx [file pone.0288285.s005.docx]

RECOMMEDATION: Major revision

Review comments to the AUTHORS:

- Abstract: JBI?
- Abstract: Why is it that the authors have recommended that the potential risks of steroids should be carefully considered? What findings from their review have led to this recommendation?
- Introduction-Line 76-current corona virus: Be more specific.
- Methods: PICOT?
- Line 140: Delete “Surely,”.
- Line 150: Is “abbreviation” a type of publication?
- Line 163: Pregnancy? Pregnant?
- Describe/define “mild”, “moderate” and “severe” COVID-19 patients either in the ‘introduction’ section or preferably in the ‘methods’ section as it had a bearing in the inclusion/exclusion criteria formulated by the authors of the present review.
- Line 189: When was the search performed?
- Line 199: PICO?
- Table 2 and Line 189: PubMed, PubMed Central and EMBASE are mentioned (Line 189). MEDLINE features in Table 2.
- Line 259: Research? Review?
- Lines 263-270, Ethical consideration: Not applicable; Delete.
- Lines 275-277: Why should there be a difference in the number of items identified by two different researchers/reviewers when the same search string is applied at the same database on the same day? This is not acceptable and points towards lapses in the search strategy. Screening of the identified items is done by 2 reviewers and discrepancies sorted out in consultation with a third reviewer. Identification of items does not require 2 reviewers. Isn’t it? And why is there a discrepancy in the numbers related to identification of items?
- Line 280: The total of “8767” records identified is wrong. Why are the numbers from PubMed added twice? The same is the case for PubMed Central and EMBASE. This is not appropriate.
- The quality of the figure related to the search strategy (PRISMA flowchart) is not good and should be replaced with a better-quality figure/picture. For related details, refer to the instructions to authors on the journal website.
- PRISMA flowchart: What is the difference between “Studies included in the review n=21” and “Reports of included studies n=21”?
- PRISMA flowchart: Registers (n=8767)? Registers (n=0)?
- PRISMA flowchart: The number of items/records identified is incorrect for reasons mentioned before (comment related to Lines 275-277).
- PRISMA flowchart: Records sought for retrieval (n=?)?
- PRISMA flowchart, Identification: The number of records/items identified from citation searching is not mentioned. Again, there are two different numbers mentioned for two different reviewers in the text (Line 278).
- S2 Figure is of poor quality and should be replaced with a better-quality one.
- Lines 284-285: Cite all the 10 RCTs.
- Lines 284-285: Cite all the 11 quasi-experimental studies.
- Line 289: Cite all the 21 studies included in the present systematic review.
- Line 354: “In the trial of (Singh, Jain and Parveen, 2021)”?
- Covid-19 vaccination: Why is this considered as a treatment strategy in the present systematic review? It shouldn’t be included. The related studies that the present authors have included have studied the occurrence of anosmia in COVID-19 vaccinated individuals infected with COVID-19.
- Line 503: Replace “Discussion on main findings” with “Discussion”.
- Line 509: Cite the previous systematic reviews and meta-analyses that are here referred to by the present authors. Besides, mention how was the present systematic review different in terms of the different parameters listed by the present authors. A Table depicting the list of related previous systematic reviews (plus the present systematic review) and the differences should be included. This is important to highlight the fact that the present systematic review is not redundant and the importance of publishing the present systematic review.
- Lines 603-609: There is a combination of Vancouver and Harvard referencing styles. Here and elsewhere, corrections should be made sticking to the Vancouver style.
- Lines 645-646: On what basis is it recommended that the potential risks of steroids should be carefully considered? What findings in the present systematic review have led the present authors to stating this recommendation?
- Line 669: Delete “desperately”.
- Lines 692-696 – Administrative information: Mention who is the student awarded the Master’s degree and the administrative roles of all others included as co-authors. Hasn’t the supervisor fulfilled the authorship criteria? The supervisor seems to be acknowledged here.

Reviewer comments to the EDITOR:

- Refer to the reviewer comments to the authors.
- The number of records in relation to identification of records/items should be corrected without fail before acceptance of this manuscript. If not, this manuscript should not be accepted for publication.
